# Solid Pseudopapillary Neoplasm of the Pancreas with High-Grade Malignant Transformation Involving p16-RB Pathway Alterations

**DOI:** 10.1155/2020/5980382

**Published:** 2020-01-13

**Authors:** Kodai Tomioka, Nobuyuki Ohike, Takeshi Aoki, Yuta Enami, Akira Fujimori, Tomotake Koizumi, Tomokazu Kusano, Koji Nogaki, Yoshihiko Tashiro, Yusuke Wada, Tomoki Hakozaki, Hideki Shibata, Takahito Hirai, Tatsuya Yamazaki, Koichiro Fujimasa, Tomoko Norose, Tomohide Isobe, Masahiko Murakami

**Affiliations:** ^1^Division of Gastroenterological and General Surgery, Department of Surgery, Showa University, Shinagawa, 1-5-8 Hatanodai, Shinagawa, 142-8666 Tokyo, Japan; ^2^Department of Pathology and Laboratory Medicine, Showa University Fujigaoka Hospital, 1-30 Fujigaoka, Aoba-Ku, Yokohama, 227-8501 Kanagawa, Japan

## Abstract

Solid pseudopapillary neoplasm (SPN) of the pancreas has generally been regarded as a low-grade malignant tumour that preferentially develops in young women and can have a good prognosis with surgery. Among the few patients who have died from metastatic SPN are mostly those whose tumours harbour an undifferentiated component characterized by diffuse sheets of cells with increased nuclear atypia and proliferative index. We herein report a case of an aggressive, fatal, solid pseudopapillary neoplasm (SPN) of the pancreas in a 63-year-old woman complaining of epigastric pain. Despite having undergone surgical resection for a 10 cm pancreatic mass and multiple liver metastases, the patient later died due to uncontrollable metastases 36 months after the initial surgery. Histological examination showed that the tumour displayed unusual high-grade malignant features, showing diffuse sheets of cells with increased nuclear atypia and proliferative activity, along with conventional low-grade malignant features. The tumour was subsequently recognized as an SPN with foci of high-grade malignant transformation according to the 2010 World Health Organization classification. Immunohistochemical studies revealed that p16-RB pathway alterations contributed to the high-grade malignant transformation. The present case report suggests the necessity for developing diagnostic and treatment methods targeting p16 and RB for high-grade variants of SPN.

## 1. Introduction

Solid pseudopapillary neoplasm (SPN) of the pancreas, a rare type of tumour accounting for 0.9%-2.7% of all pancreatic tumours [1], has generally been regarded as a low-grade malignant tumour that preferentially develops in young women and can have a good prognosis with surgery. Such tumours histologically comprise poorly cohesive epithelial cells forming solid and pseudopapillary structures. Only a few patients have died from metastatic SPN—mostly those whose tumours harbour an undifferentiated component characterized by diffuse sheets of cells with increased nuclear atypia and proliferative index [2, 3]. Such high-grade tumours have been subclassified as SPN with foci of high-grade malignant transformation.

We herein report a case involving such an aggressive SPN with a rapid and fatal clinical course and discuss its molecular events and malignancy.

## 2. Case Presentation

### 2.1. Clinical Course

**Figure 1:**
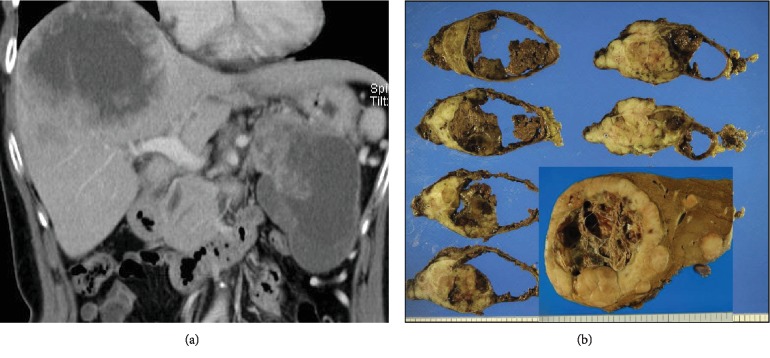
Computed tomography (CT) images and surgical specimens. (a) Abdominal CT showing similar large solid masses with cystic components in the pancreatic tail and right hepatic lobe. (b) Macroscopic cross-section of the pancreatic tumour revealing a bulky mass (*φ*10 cm) that consists of a cystic component containing extensive haemorrhagic, friable necrotic debris, and a white-to-grey solid component showing lobular growth. Liver metastatic foci also show similar macroscopic findings (inset).

The patient was a 63-year-old woman complaining of epigastric pain. Physical examination showed no significant abnormal findings; laboratory data were normal except for the slightly elevated *γ*-glutamyl transpeptidase (320 IU/l). Moreover, although several serum tumour markers, such as carcinoembryonic antigen, carbohydrate antigen 19-9, alpha-fetoprotein, and protein induced by vitamin K absence II (PIVKA-II), were all within normal ranges, neuron-specific *γ*-enolase (21 ng/ml) and carbohydrate antigen 125 (118 U/ml) were mildly elevated. Gastrointestinal endoscopy showed no significant abnormalities. Abdominal imaging (e.g., computed tomography and ultrasonography) revealed similar large solid masses with cystic components in the pancreatic tail (as a single lesion) and right hepatic lobe (as multiple lesions) (Figure 1(a)). Endoscopic retrograde cholangiopancreatography showed discontinuation of contrast agent inflow into the pancreatic body. A liver biopsy suggested pancreatic SPN, revealing solid sheets or nests of uniform, poorly cohesive monomorphic cells with round-to-oval nuclei and eosinophilic cytoplasm with the nuclear and cytoplasmic immunohistochemical expression of *β*-catenin (17C2, Leica, Newcastle upon Tyne, UK) in addition to positive findings for vimentin (V9, Leica) and CD56 (CD564, Leica) and negative findings for CKAE1/AE3 (AE1 and AE3, Leica), chromogranin A (5H7, Leica), and trypsin (MAB1482, EMD Millipore, Billerica, MA, USA) (immunohistochemical staining was performed using the avidin-biotin complex detection method with a BenchMark Automated Immunostainer; Ventana Medical Systems, Inc., Tucson, AZ, USA).

Considering the diagnosis of pancreatic SPN with multiple liver metastases, distal pancreatectomy and splenectomy with lymph node dissection were performed, followed by percutaneous transhepatic portal embolisation and right hepatic lobectomy. No adjuvant therapy was provided. After 7 months, however, recurrent liver metastases were revealed through computed tomography, for which partial liver resection was performed. Nonetheless, the patient died of uncontrollable multiple metastatic lesions spreading throughout the liver 36 months after the initial surgery.

### 2.2. Pathological Findings

Grossly, the cross-section of the pancreatic tail tumour revealed a bulky mass measuring 10 × 7 cm that consisted of a cystic component containing extensive haemorrhagic, necrotic debris and a solid component showing lobular growth (Figure 1(b)). Liver metastatic foci (maximum size of 13 × 12 cm) also showed similar macroscopic findings.

**Figure 2:**
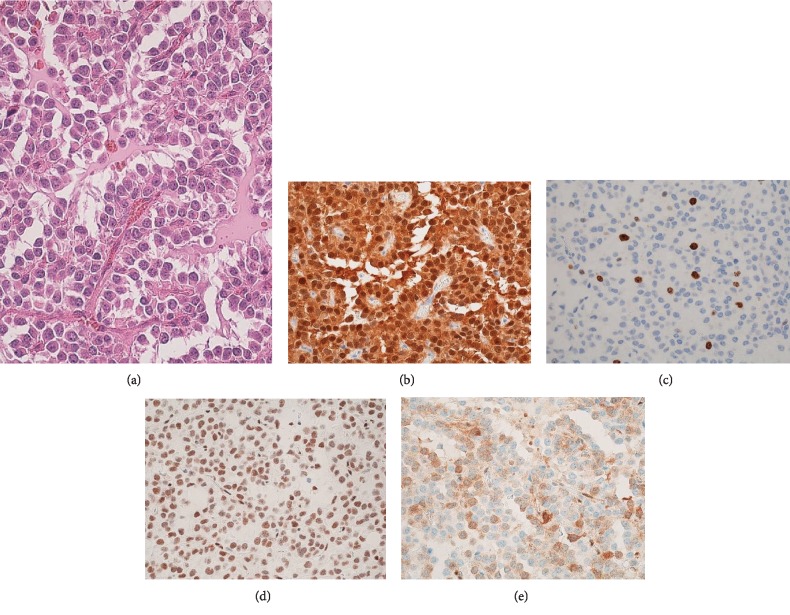
Histological and immunohistochemical findings of the low-grade pseudopapillary areas of the pancreatic tumour (×400). (a) Conventional pseudopapillary structures composed of uniform, poorly cohesive monomorphic cells and fibrovascular stalks. Mitosis was not prominent. (b) Nuclear and cytoplasmic immunohistochemical expression of *β*-catenin. (c) Ki-67 index: 6%. (d) Normal nuclear staining for RB protein. (e) Heterogeneous staining for p16 protein.

**Figure 3:**
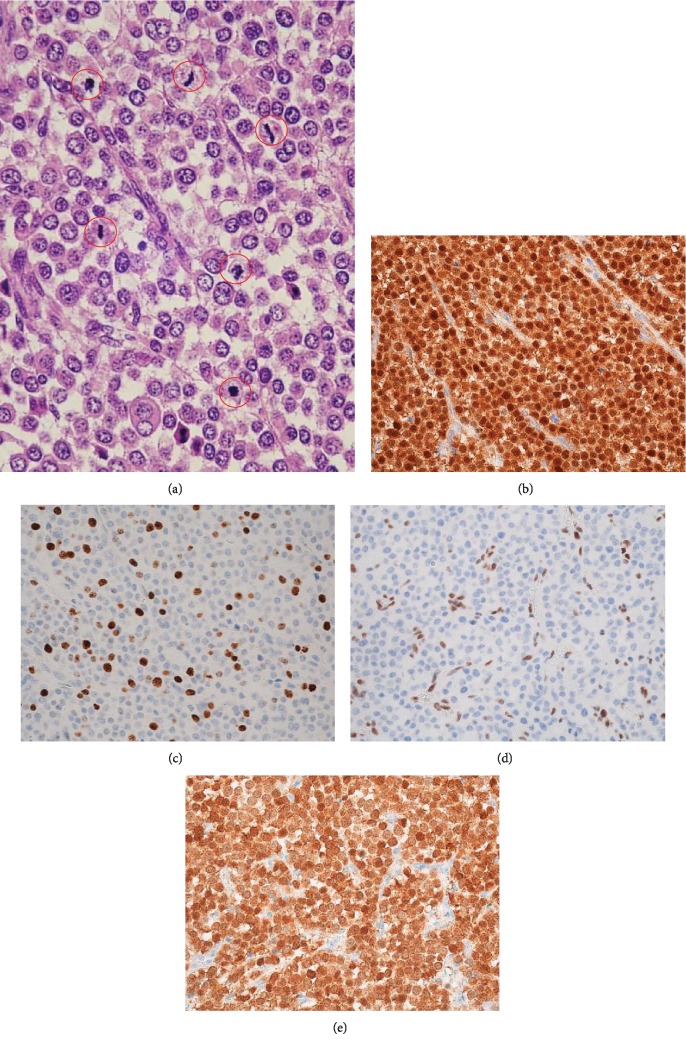
Histological and immunohistochemical findings of high-grade undifferentiated areas of the pancreatic tumour (×400). (a) Nested-to-diffuse growth of more poorly cohesive monomorphic cells with increased nuclear atypia. Mitoses were conspicuous (indicated by red circles). (b) Nuclear and cytoplasmic immunohistochemical expression of *β*-catenin. (c) Ki-67 index: 22%. (d) Diffuse RB protein loss. (e) Diffuse p16 protein overexpression.

Histologically, half of the primary tumour showed conventional pseudopapillary structures composed of uniform, poorly cohesive monomorphic cells and fibrovascular stalks in which mitosis was not prominent, with a Ki-67 (MMI, Leica) index of 6% (Figure 2). In contrast, the remaining lobular solid areas extended towards the surroundings of the primary lesion, while all liver metastatic lesions showed nested-to-diffuse growth of more poorly cohesive monomorphic cells with increased nuclear atypia, in which mitoses were conspicuous (≥4 per high-power field (HPF)) and the Ki-67 index was 22% (Figure 3). All lesions showed typical immunohistochemical features for SPN, such as nuclear and cytoplasmic expression of *β*-catenin, positive findings for vimentin and CD56, and negative findings for chromogranin A. Additional immunohistochemical staining using antibodies for RB (13A10, 1 : 100 dilution), p16 (E6H4TM; Ventana), and p53 (DO-7; Ventana) showed that the pseudopapillary structure areas with lower proliferative activities had normal staining for RB protein (in the nucleus) and heterogeneous staining for p16 protein (in the nucleus and cytoplasm) and p53 protein (in the nucleus) (Figure 2), while the undifferentiated diffuse sheet areas with higher proliferative activities showed a diffuse loss of RB protein, diffuse overexpression of p16 protein, and heterogeneous staining for p53 protein (Figure 3).

### 2.3. Comparison with Conventional SPNs

As a comparison study, conventional SPNs (*n* = 5) were immune-stained for Ki-67, RB, p16, and p53. The results showed that all conventional SPNs had a very low Ki-67 index (<3%), normal staining for RB protein, scant (*n* = 2) or heterogeneous (*n* = 3) staining for p16 protein, and heterogeneous staining for p53 protein.

## 3. Discussion

Majority of SPNs (conventional SPNs) are low-grade malignant tumours that show an excellent long-term prognosis for localized or even metastatic or recurrent disease after complete surgical resection [4, 5]. However, as in the present case, a few patients have died from metastatic SPN, mostly those whose tumours harbour an amorphous, undifferentiated component lacking typical pseudopapillary structures [3, 6, 7]. Such fatal tumours have been subclassified as SPN with foci of high-grade malignant transformation, which is histologically characterized by diffuse sheets of cells with increased nuclear atypia, abundant mitoses, necrosis, and rarely sarcomatous changes. The tumour identified in the present case seems to be consistent with this rare variant.

Conventional SPNs harbour somatic point mutations in exon 3 of CTNNB1, the gene encoding *β*-catenin, leading to abnormal nuclear localisation of the *β*-catenin protein, which can be highlighted using immunohistochemistry [2, 3]. Recently, Amato et al. [8] identified inactivating mutations in epigenetic regulators (KDM6A, TET1, and BAP1) associated with metastatic SPNs, in addition to CTNNB1-activating mutations. However, few studies have focused on investigating molecular abnormalities in high-grade malignant SPNs due to their rarity [3, 6, 7].

In the tumour identified herein, we noticed p16-RB pathway alterations in addition to *β*-catenin abnormalities. Accordingly, diffuse RB protein loss and diffuse p16 protein overexpression were found in high-grade undifferentiated areas of both the primary and metastatic lesions, while a normal staining pattern for RB protein and a heterogeneous staining pattern for p16 protein were observed in low-grade pseudopapillary areas of the primary lesion and in all conventional SPNs of the comparison cases. These results indicate multistep development involving both morphological (low-grade pseudopapillary structures to high-grade diffuse sheets) and genetic (*β*-catenin abnormalities plus changes to the p16-RB pathway) alterations. The combination of diffuse RB protein loss and diffuse p16 protein overexpression has often been found in highly aggressive malignant tumours with high proliferative activities, a finding convincingly suggestive of changes in the p16-RB pathway [9, 10]. Therefore, RB and p16 immunostaining seems to be useful for identifying the high-grade component in SPNs, while treatment targeting the p16-RB pathway may be effective for high-grade SPNs.

According to the 2017 World Health Organization classification [11], pancreatic neuroendocrine neoplasms are classified as well-differentiated neuroendocrine tumour (NET) grade (G) 1, NET G2, or NET G3 or poorly differentiated neuroendocrine carcinoma (NEC) G3 based on histological differentiation, mitotic count, and Ki-67 index. G1 is a NET with a mitotic count of <2/10 HPFs and/or a Ki-67 index of <3%; G2 is a NET with a mitotic count of 2-20/10 HPFs and/or a Ki-67 index of 3%-20%; and G3 is a NET or NEC with a mitotic count of >20/10 HPFs and/or a Ki-67 index of >20%. Although discriminating between NET G3 and NEC G3 can be difficult, NET G3 is characterized by a well-differentiated histology, a mitotic count of ≤40/10 HPFs, a Ki-67 index of <55%, an intact RB, and a median survival of several years, while NEC G3 is characterized by a poorly differentiated histology (of either small or large cell types), a mitotic count of >40/10 HPFs, a Ki-67 index of >55%, diffuse loss of RB expression, a highly aggressive malignancy, and a median survival of less than 1 year [11, 12]. Applying this grading system to the SPN identified in the present case, the high-grade undifferentiated component seems to fall under the NEC G3 category based on the poorly differentiated histology, mitotic count of ≥40/10 HPFs, and diffuse loss of RB expression, although the Ki-67 index was 22% and the survival period was 36 months. Interestingly, the low-grade pseudopapillary component, which is probably a preceding lesion of this tumour, belongs to the NET G2 category based on the organoid (well-differentiated) histology and a Ki-67 index of 6%. This implies that the SPN identified herein may have been biologically distinct from conventional SPNs at its onset given that conventional SPNs usually show a considerably low Ki-67 index of <3%, as shown in our study and several previous reports [3, 7], and belong to the NET G1 category.

In conclusion, a case of aggressive, fatal pancreatic SPN with high-grade malignant transformation involving p16-RB pathway alterations was reported. Surgery is the main and standard treatment for SPNs. In addition, it may be useful to consider this type of entity when encountering SPNs exhibiting an unusually lethal course. It was suggested that our finding could be useful in establishing further diagnostic methods and therapeutic strategies for SPNs.
