# Corrigendum to “Solid Pseudopapillary Neoplasm of the Pancreas with High-Grade Malignant Transformation Involving p16-RB Pathway Alterations”

**DOI:** 10.1155/2020/5182870

**Published:** 2020-08-26

**Authors:** Kodai Tomioka, Nobuyuki Ohike, Takeshi Aoki, Yuta Enami, Akira Fujimori, Tomotake Koizumi, Tomokazu Kusano, Koji Nogaki, Yoshihiko Tashiro, Yusuke Wada, Tomoki Hakozaki, Hideki Shibata, Takahito Hirai, Tatsuya Yamazaki, Koichiro Fujimasa, Tomoko Norose, Tomohide Isobe, Masahiko Murakami

**Affiliations:** ^1^Division of Gastroenterological and General Surgery, Department of Surgery, Showa University, Shinagawa, 1-5-8 Hatanodai, Shinagawa, 142-8666 Tokyo, Japan; ^2^Department of Pathology and Laboratory Medicine, Showa University Fujigaoka Hospital, 1-30 Fujigaoka, Aoba-Ku, Yokohama, 227-8501 Kanagawa, Japan

In the article titled “Solid Pseudopapillary Neoplasm of the Pancreas with High-Grade Malignant Transformation Involving p16-RB Pathway Alterations” [1], author Yusuke Wada was affiliated to the Division of Gastroenterological and General Surgery, Department of Surgery, Showa University, Shinagawa, 1-5-8 Hatanodai, Shinagawa, 142-8666, Tokyo, Japan, which is incorrect. The corrected list of affiliations is shown above.
